# Magnetic Cellulose-Chitosan Nanocomposite for Simultaneous Removal of Emerging Contaminants: Adsorption Kinetics and Equilibrium Studies

**DOI:** 10.3390/gels7040190

**Published:** 2021-10-30

**Authors:** Phodiso Prudence Mashile, Philiswa Nosizo Nomngongo

**Affiliations:** 1 Department of Chemical Sciences, Doornfontein Campus, University of Johannesburg, P.O. Box 17011, Doornfontein 2028, South Africa; prudencem.mashile@gmail.com; 2 Department of Science and Innovation-National Research Foundation South African Research Chair Initiative (DSI-NRF SARChI), Nanotechnology for Water, University of Johannesburg, P.O. Box 17011, Doornfontein 2028, South Africa

**Keywords:** hydrogel, swelling, propranolol, atenolol, carbamazepine, water system

## Abstract

The presence of pharmaceuticals in water systems threatens both terrestrial and aquatic life across the globe. Some of such contaminants are β-blockers and anticonvulsants, which have been constantly detected in different water systems. Various methodologies have been introduced for the removal of these emerging pollutants from different waters. Among them, adsorption using nanomaterials has proved to be an efficient and cost-effective process for the removal of pharmaceuticals from contaminated water. In this this study, a firsthand/time approach applying a recyclable magnetic cellulose-chitosan nanocomposite for effective simultaneous removal of two β-blockers (atenolol (ATN)) and propranolol (PRP) and an anticonvulsant (carbamazepine (CBZ)) is reported. A detailed characterization of the eco-friendly, biocompatible cellulose-chitosan nanocomposite with magnetic properties was performed at various rates of synthesis using X-ray diffraction (XRD), Brunauer-Emmett-Teller (BET), and Fourier transform infrared (FTIR) spectroscopy. A N_2c_ adsorption-desorption test showed that the prepared nanocomposite is mesoporous, with a BET area of 112 m^2^ g^−1^. The BET isotherms results showed that the magnetic cellulose-chitosan nanocomposite has a pore size of 24.1 nm. The adsorption equilibrium of PRP and CBZ fitted with the Langmuir isotherm was consistent with the highest coefficient of determination (R^2^ = 0.9945) and (R^2^ = 0.9942), respectively, while the Sips model provided a better fit for ATN, with a coefficient of determination R^2^ = 0.9956. The adsorption rate was accompanied by a pseudo-second-order kinetics. Moreover, the swelling test showed that up to 100 percent swelling of the magnetic cellulose-chitosan nanocomposite was achieved.

## 1. Introduction

The presence of pharmaceutical residues in water systems has received great attention because they have been acknowledged as emerging environmental pollutants [1]. Pharmaceuticals such as β-blockers and anticonvulsants are found in water systems because they are very popular drugs that are used worldwide. β-Blockers such as propranolol (PRP) and atenolol (ATN) are cardioselective β1-adrenergic receptor blocking drugs used to treat hypertension, prevent angina pectoris, treat arrhythmia, and lessen the risk of heart problems after a heart attack [2,3]. Carbamazepine is usually used as an anticonvulsant for the treatment of epilepsy, bipolar disorder, mental illnesses, schizophrenia, depression, seizure disorders and relief of neuralgia [4]. These drugs are partially excreted unaltered after ingestion and enter the environment through a variety of channels, including household waste, hospital discharges, and improper manufacturer disposal into wastewater treatment plants [5,6,7]. On average, hospital effluents had a greater detection frequency and concentration of these drugs than residential waste [7]. Literature reports that the removal efficiencies of ATN, CBZ and PRP by conventional wastewater treatment plants (WWTPs) are often less than 10% [8]), this is because most of the WWTPS are not designed to eliminate pharmaceutical residues [4,9]. Consequently, various pharmaceuticals are released into the receiving nearby rivers, which has a potential of causing adverse health effects to aquatic and terrestrial life. It is therefore critical to efficiently remove these β blockers and anticonvulsants from the environment to guarantee safe discharge [7].

Elimination of ATN, CBZ and PRP using adsorption technology based on the use of nanomaterials as adsorbent has received more attention in recent years [4,10,11]. Nano-adsorption technology offers advantages such as cost reduction, versatility, high removal efficiency, short cleanup time and the possibility of regeneration/reuse of spent adsorbent [12,13]. The use of biopolymers as adsorbent materials in multidisciplinary fields is currently under study and they have received great attention as environmentally-friendly sorbents for adsorptive water treatment [14].

Recently, research interest has shifted to the design and production of new adsorbent materials for the removal of pharmaceutical residues in water systems that can deliver cost-effective and efficient adsorption technologies. These materials should provide excellent properties such as superior adsorption capacity, recyclability, stability and separability [15,16,17]. Hydrogels appear to be an effective adsorbent for the treatment of various aqueous pollutants [14,18]. This is due to their attractive features such as 3D network structures, high adsorption capacity, large surface area, hydrophilicity and multiple functional groups [19]. In water treatment systems, hydrogels are very efficient for the trapping of a wide range of organic and inorganic aqueous contaminants, including metal ions, harmful dyes and lethal pharmaceutical waste [14,20,21]. Different basic materials are used for the synthesis of hydrogels, including silica, glass beads, chitosan, cellulose, polyacrylic acid polyester, to name a few that are interlinked through different chemical (thermal, photo or radiation induced) or physical pathways that give rise to a three-dimensional gel network [22]. The crosslinker also gives the parent polysaccharide resistance characteristics for lower pH solutions and higher temperature ranges. The key drawback in synthesis is the decrease in the number of sorption sites, with the rise in density of the crosslinker in crystalline domains, which then distorts the polymer matrix’s original crystal structure [14,23,24].

Cellulose and chitosan are two biodegradable biomaterials used in the production of hydrogels. The incorporation of cellulose during the synthesis of hydrogels, contributes to improve the mechanical properties of the adsorbent due to its rigid molecular chains [19,25]. However, its poor recovery performance and low adsorption capacity limit the application of cellulose for the adsorptive removal of various pollutants [26]. Chitosan contains amino groups that allow the adsorption to wide range of pollutants. However, chitosan is known to have very poor mechanical strength which limits its application [19,27]. To overcome these limitations, chitosan is usually combined with cellulose to produce an excellent hydrogel material with improved properties.

Hydrogels containing cellulose and chitosan-based products have been reported to be biocompatible, showing better adsorption capability, and significant improvement in pH sensitivity and mechanical properties [28,29,30].

Therefore, the current study focuses on the synthesis, characterization, and application of magnetic cellulose-chitosan hydrogel nanocomposite for the removal of β blockers and anticonvulsants from wastewater. Magnetic nanoparticles were incorporated into a cellulose-chitosan hydrogel to improve separability and recyclability of the adsorbent from aqueous solutions. The adsorption mechanism, percentage swelling, kinetics, isotherms models and regeneration were investigated. The effect of sample pH, initial concentration, contact time and mass of adsorbents was optimized using univariate and multivariate approaches.

## 2. Results and Discussion

### 2.1. Characterization

#### 2.1.1. Fourier Transform Infrared Spectroscopy

**Figure 1:**
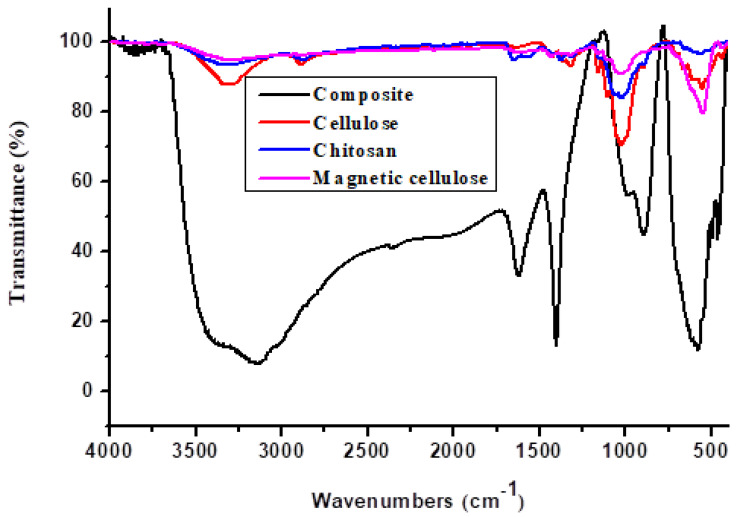
FTIR image showing magnetic cellulose-chitosan composite, cellulose, chitosan and magnetic cellulose.

The FTIR spectrum of the magnetic cellulose-chitosan suggested that Fe_3_O_4_ is effectively incorporated into chitosan and cellulose (Figure 1). This is due to the presence of a sharp peak at 578 cm^−1^ which is usually attributed to the Fe-O bond [31]. The FTIR spectrum of the nanocomposite (Figure 1) revealed a broad peak between 3438 cm^−1^ and 3632 cm^−1^ which was assigned to the stretching vibration bands of O-H and N-H (amide) groups [32]. These peaks confirmed that the magnetic hydrogel nanocomposite was composed of cellulose and chitosan [32]. The shoulder peak 3436 cm^−1^ in Figure 1 was assigned to the distinctive absorption bands of the N-H (amine) bond of chitosan, which overlapped with the stretching vibrations of O-H of MCC and pristine cellulose in the magnetic cellulose-chitosan nanocomposite (Figure 1) [32]. The bands around 1701 and 1489 cm^−1^ in the composite and chitosan were ascribed to C=O stretching vibration of amide groups and the asymmetric stretching of carboxyl groups [31]. Finally, the peaks around 1000 cm^−1^ in the composite, chitosan, cellulose and magnetic cellulose in (Figure 1) were assigned to the C-O stretching vibration of C-OH in chitosan and cellulose [33]. These findings suggest that the magnetic cellulose-chitosan hydrogel nanocomposite was indeed generated via the interaction between the N-H groups of chitosan and the hydroxide groups of MCC [32]. These modifications supported the theory that the Fe_3_O_4_ particles were coated with chitosan and cellulose after preparation. Furthermore, these results agreed with those reported in previous studies [32,33].

#### 2.1.2. X-ray Diffraction Spectroscopy

**Figure 2:**
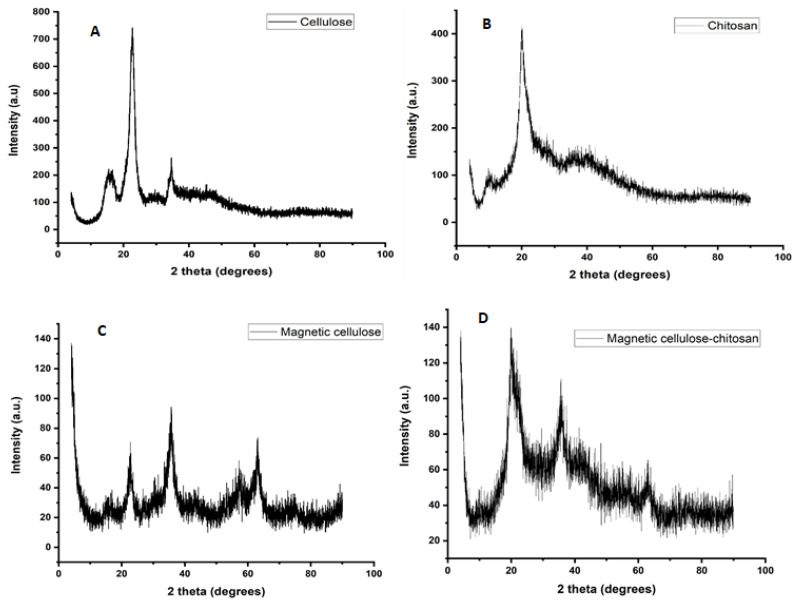
XRD patterns of (**A**) pristine cellulose, (**B**) magnetic cellulose, (**C**) pristine chitosan and (**D**) magnetic cellulose-chitosan nanocomposite.

The XRD patterns of the pristine cellulose, magnetic cellulose, pristine chitosan and magnetic cellulose-chitosan nanocomposite are illustrated in Figure 2. The peaks between 15° and 34° in Figure 2A were ascribed to the crystalline structures of pristine cellulose [34,35]. In the XRD pattern of pristine chitosan (Figure 2C), the reflection peak around 20° were attributed to the crystallinity of chitosan [33]. However, the peak between 21° and 22° in Figure 2D has increased from (Figure 2B) due to the addition of chitosan, while the magnetite phase appears to have shifted or disappeared with the addition of chitosan. This is because Fe_3_O_4_ interacted with –NH_2_/OH chitosan groups and produced cationic amine-rich Fe_3_O_4_ particles, which were coated by chitosan during the coating process [33]. According to previous authors, these results demonstrate that crystallinity of the magnetic cellulose-chitosan nanocomposite was suppressed by strong interaction between the magnetic nanoparticles and O–C and N–C bonds of cellulose and chitosan [36].

#### 2.1.3. Transmission Electron Microscopy

**Figure 3:**
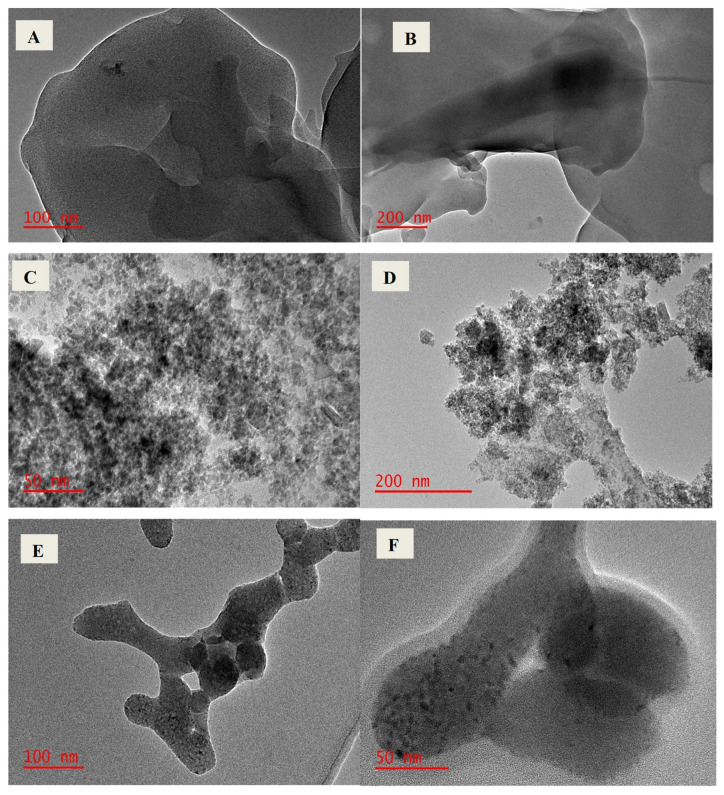
TEM images of (**A**) pristine cellulose, (**B**) pristine chitosan, (**C**) magnetic cellulose and (**D**–**F**) magnetic cellulose-chitosan hydrogel (at scales i.e., 50 nm, 100 nm, and 200 nm.

Morphological properties of the magnetic cellulose-chitosan hydrogel nanocomposite were studied by the transmission electron microscopy (TEM) technique. The TEM images of pristine cellulose, pristine chitosan, magnetic cellulose and magnetic cellulose-chitosan hydrogel nanocomposite are presented in Figure 3. In comparison with pristine cellulose and chitosan, the appearance of tiny black particles on the TEM images of magnetic cellulose and magnetic cellulose-chitosan (Figure 3D–F) demonstrates that magnetic particles were incorporated in the structural matrices. In addition, the introduction of chitosan to the magnetic cellulose (Figure 3C) resulted in noticeable morphological changes which confirm the formation of hydrogel composite (Figure 3E,F).

#### 2.1.4. Magnetization Analysis

**Figure 4:**
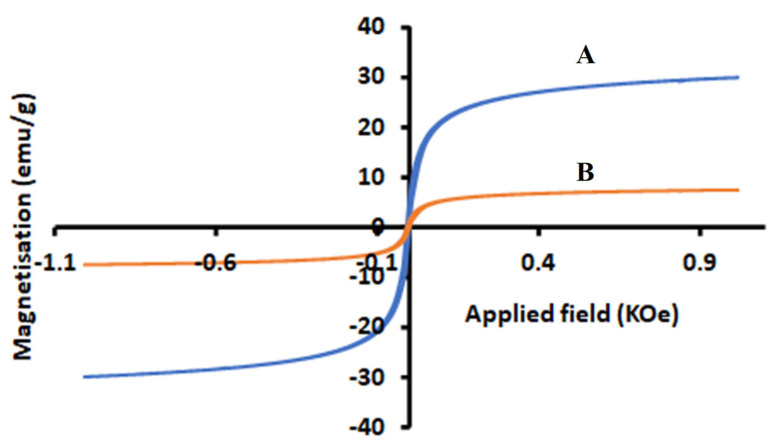
Magnetic properties of (**A**) magnetic cellulose and (**B**) magnetic cellulose-chitosan hydrogel nanocomposite.

The magnetic properties of magnetic cellulose and magnetic cellulose-chitosan hydrogel nanocomposite was examined by vibrating sample magnetometry (VSM). The results obtained are presented in Figure 4. Based on Figure 4, the saturation magnetization values of the magnetic cellulose and magnetic cellulose-chitosan hydrogel nanocomposite were 30 and 7.48 emu·g^−1^, respectively. As seen in Figure 4B, the magnetic property of the nanocomposite has decreased significantly in comparison to magnetic cellulose during the incorporation of chitosan. However, the magnetization value of the nanocomposite is enough for magnetic separation via the application of an external magnetic field. Similar results have been reported in the literature [34].

#### 2.1.5. N_2_ Adsorption-Desorption Analysis

**Figure 5:**
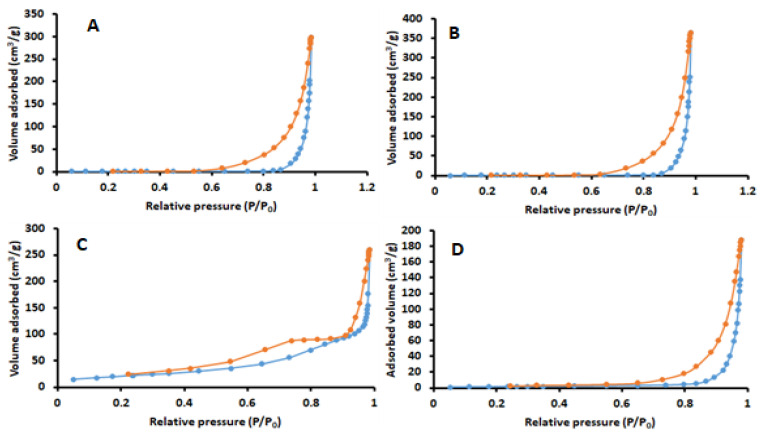
BET N_2_ adsorption-desorption isotherms of (**A**) pristine cellulose, (**B**) pristine chitosan, (**C**) magnetic cellulose and (**D**) magnetic cellulose-chitosan hydrogel nanocomposite.

The porosity properties of the nanocomposite were investigated using Brunauer-Emmett-Teller (BET) N_2_ adsorption-desorption measurements. The BET N_2_ adsorption-desorption isotherms of pristine cellulose, pristine chitosan, magnetic cellulose and magnetic cellulose-chitosan hydrogel are illustrated in Figure 5. According to the IUPAC classification, the N_2_ adsorption-desorption isotherms of the samples were categorized as type I/IV [37] with hysteresis loops. These results revealed that the samples display a characteristic mesoporous structure [37]. The surface properties of the materials are presented in Table 1.

### 2.2. Percentage Swelling Ratio of the Hydrogel

**Figure 6:**
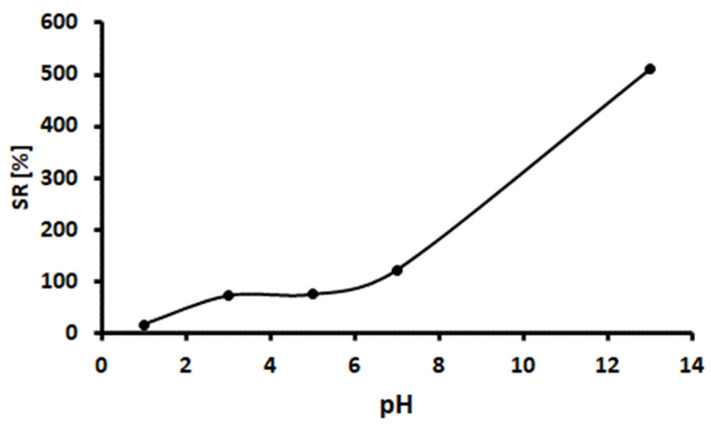
Hydrogel tests pH-sensitive swelling percentage of magnetic cellulose-chitosan hydrogel nanocomposite.

The magnetic cellulose-chitosan nanocomposite hydrogel displayed more than 100 percent swelling ratio (%SR), with swelling increasing over time, at first rapidly and later slowly. Figure 6 shows that the magnetic cellulose-chitosan hydrogel nanocomposite’s water uptake ability increased with the rise in pH. The swelling is due to water absorption by pores of the adsorbent. The swelling percentage slowly increased to a balanced state following the rapid swelling process. There is an opposite elasticity strength that balance the network stretch and prevents its deformation against a favorable osmotic force during a hydrogel swelling [38]. Further swelling is avoided by elasticity and osmotic forces. At alkaline medium (pH 13.0), the hydrogels displayed the highest swelling percentage.

### 2.3. Optimization of the Adsorption Process

**Figure 7:**
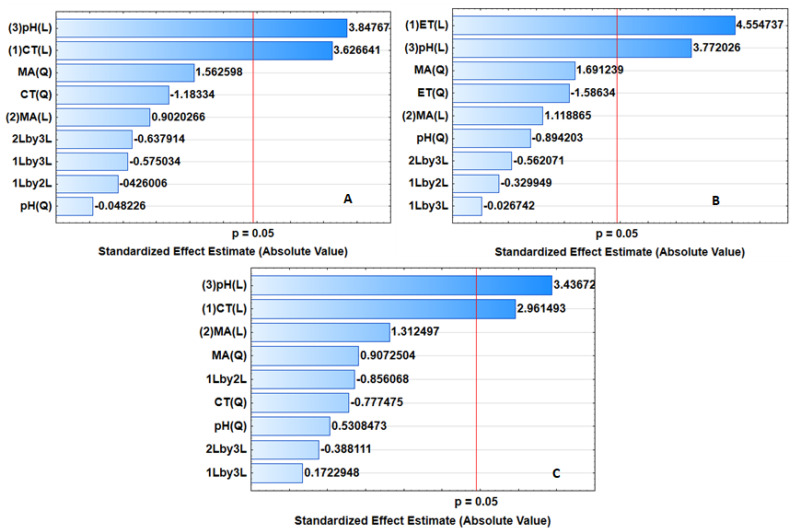
Pareto chart for standardized effects for adsorptive removal of (**A**) ATN, (**B**) PRP and (**C**) CBZ.

A central composite design (CCD) was used to investigate the effect of the independent variables on the analytical response. These factors included sample pH, mass of adsorbent (MA) and contact time (CT). Table S1 presents the CCD matrix and the experimental data. The experimental data was further explained using analysis of variance (ANOVA). The ANOVA results as represented in the form of Pareto charts (Figure 7) were used to identify the most important variables and their interactions. This means that the factor or interaction is statistically significant if the bar length crosses the red line at a confidence level of 95% (*p*-value = 0.05). Figure 7A–C show that the sample pH and contact time were significant at the 95% confidence level, while the mass of the adsorbent was not significant at the 95% confidence level.

#### 2.3.1. Response Surface Methodology

**Figure 8:**
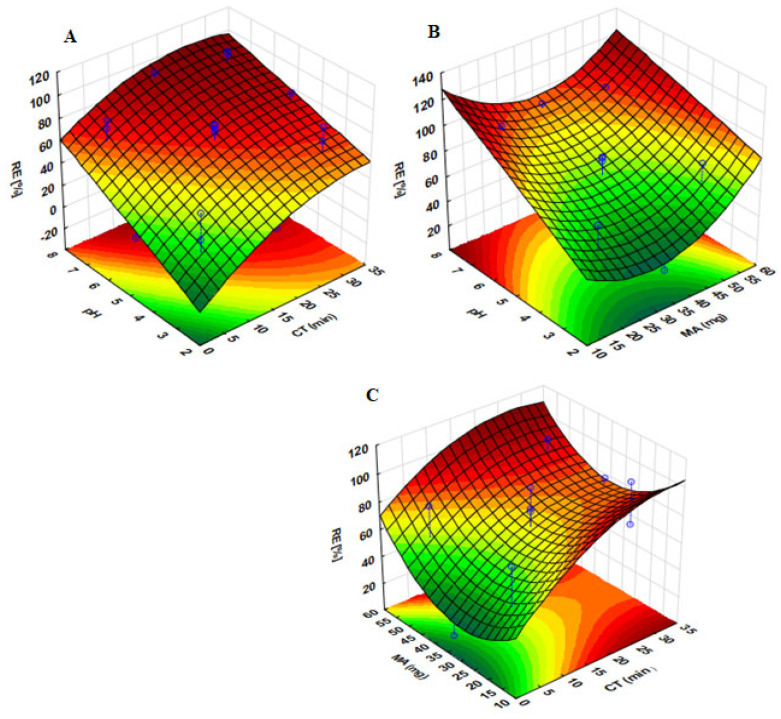
3-D surface plots for combined effects of the independent factors on the optimization of ultrasound assisted adsorption process. (**A**) Interactions between sample pH and contact time (CT) while MA is fixed at 35 mg; (**B**) interaction between sample pH and mass of adsorbent (MA) while CT is fixed at 17.5 min, (**C**) interaction between MA and CT while pH is fixed at 7.

The interactive effects of the independent variables while one of the parameters are fixed at its central point (Table S1) were further investigated using 3D response surface plots (Figure 8). Figure 8A demonstrates that when the mass of adsorbent is fixed at 35 mg (central point, see Table S1), the increase in contact time and sample pH led to an increase in the analytical response (%removal efficiency (%RE)) up to 95%. The surface charge of sorbent and ionization of the analytes are predominantly driven by sample pH. Atenolol, CBZ, and PRP are basic analytes that have pKa values of 9.6, 13.6 and 9.5, respectively. Figure 8B indicates that as the pH of the solution changes from 5 to 8 and the mass of adsorbent is increases, the analytical response also increases. This suggests that the species of these analytes at sample pH values ranging from 5 to 8 are predominately positively charged and the surface of the adsorbent was becoming more negatively charged, thus, promoting the favorable adsorption process of the analytes on the surface of the adsorbent through electrostatic interactions. Additionally, Figure 8B,C reveal that even though mass of adsorbent was not statistically significant (according to Figure 7), its increase led to a high %RE. The increasing %RE with respect to MA and CT (Figure 8C) can be attributed to the higher surface area and increased number of vacant sites, as well as the longer interaction time. Furthermore, these findings revealed that the use of ultrasonic power enhanced the removal efficiency. This is because of the physical phenomenon of acoustic cavitation, which involves the development, growth and collapse of micrometrical bubbles caused by the transmission of a pressure wave in a liquid. The ultrasound waves increase the mass transfer by convection and reactivation of the adsorbent surface [39]. Shock waves can cause microturbulence in the interfacial layers that surround nearby solid particles [40]. Ultrasonic sample irradiation improves mass transfer processes by weakening the affinity between adsorbate and adsorbent [39,41].

#### 2.3.2. Desirability Function

**Figure 9:**
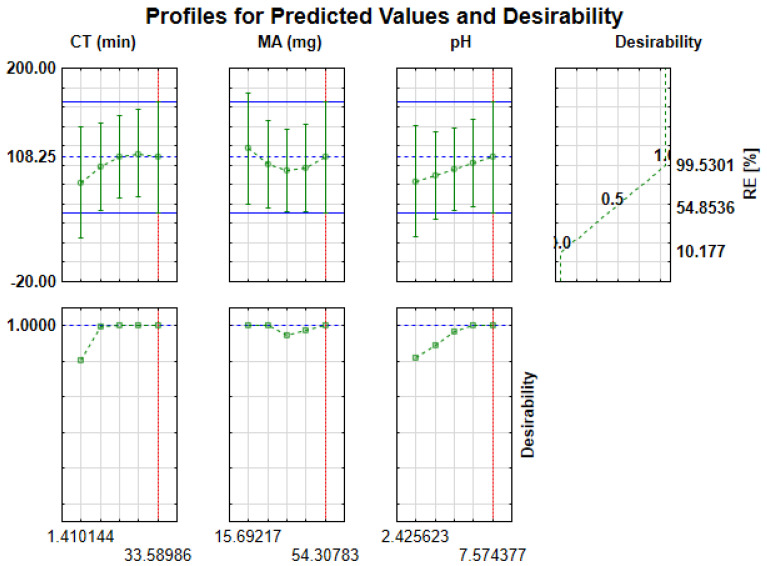
Profile for predicated percentage removal efficiency (%RE) values (**top graphs**) and desirability function (**bottom plots**) for removal of ATN, CBZ and PRP. Dashed horizontal lines (**top plots**) and vertical lines (**bottom plots**) indicated predicted %R and optimum values for each independent factor, respectively. The dashed horizontal lines in bottom plots represent desirability function/score.

The TIBCO^®^ Statistica™ package version 13 (StatSoft, Palo Alto, CA, USA) allowed the application of a desirability function to obtain the optimal conditions for sample pH, MA and CT (Figure 9). The desirability values in Figure 9 (top right hand side) representing the minimum, middle and maximum were aligned as 0.0 (undesirable), 0.5 and 1.0 (very desirable), respectively [40]. According to Figure 9 (top right-hand side), the desirability scores of 1.0, 0.5 and 0.0 gave removal efficiencies of 99.5%, 54.8 and 10.2%, respectively. The maximum desirability value closer to 1.0 (very desirable) implies that the corresponding variable condition is at its optimum level (Figure 9, bottom left-hand side). In addition, the vertical lines observed in Figure 9 (top and bottom left-hand side) represent the optimum predicted values for each variable. In this study, the desirability value of 1.0 was chosen as the target value used to attain optimum conditions. Figure 9 (top left-hand side) display the changes in the level of each experimental variable, analytical response, and overall desirability (bottom left-hand side). Therefore, based on the desirability score of 1.0, the percentage removal of target analytes was optimized at 108% and the optimum conditions were 34 min, 54 mg and 7 for contact time, mass of adsorbent and sample pH, respectively. Experimentally the optimum conditions were tested and %RE values greater than 99.4 ± 2.5% were obtained. These findings were consistent with the predicted values at 108% confidence level.

### 2.4. Equilibrium Isotherms

Table 2 illustrates the summary of the constants and coefficient of determination (R^2^) values obtained from the two and three-parameter isotherms applied for the adsorption of PRP, ATN and CBZ onto the surface of the magnetic cellulose-chitosan nanocomposite. A Langmuir equation fitted better with the highest coefficient of determination (R^2^ = 0.9945) value for PRP and R^2^ = 0.9942 for CBZ, therefore it provides a better fit for the experimental data, while the Sips model provides a better fit for ATN, with a correlation coefficient of R^2^ = 0.9956. The Langmuir model assumes that uniform adsorption sites are distributed on the adsorbent surface and that only a single layer adsorption occurs [42], which is consistent with the experimental data. The Sips model is based on the Langmuir model and the Freundlich model [42], and can be used to account for the heterogeneity of the system as it reduces the Freundlich isotherm at low adsorbate concentrations, while it also reduces the Langmuir model at high concentrations when the exponent is close to the unity [43]. The Redlich-Peterson equation also fitted the data well, although the coefficient of determinations were slightly lower (0.9917 < R^2^ < 0.9936). The Freundlich model was not satisfactory in this study, presenting the lowest correlation (0.9208 < R^2^ < 0.9877). The results suggest that the reaction process and mechanisms of CBZ, PRP, and ATN adsorption onto magnetic cellulose-chitosan nanocomposite are consistent with single-layer adsorption at low concentrations and multilayer adsorption at high concentrations. These results are consistent with those from literature [42]. The overall adsorption data followed the order Sips > Langmuir > Redlich-Peterson > Freundlich for ATN and Langmuir > Redlich-Peterson > Sips > Freundlich for PRP and CBZ.

### 2.5. Adsorption Kinetics

The PRP, ATN and CBZ adsorption kinetics on nanocomposite-based magnetic cellulose chitosan were studied using two kinetic models. The kinetic data was subjected to the linear forms of the pseudo-first and the pseudo-second order models presented in Table 3. The values of q_e_, k_1_ and k_2_ in Table 3 were calculated from the slope and intercept of the plots of ln(q_e_ − q_t_) or 1/q_t_ vs. t, respectively. The results in Table 3 revealed that the calculated q_e_ for all the analytes using the pseudo-first order model was lower than the experimental q_e_. Moreover, the R^2^ values ranged from 0.8720–0.8812 and they were much lower than those obtained using pseudo-second order model. These findings suggest that the adsorption process of PRP, ATN and CBZ onto magnetic cellulose-chitosan nanocomposite is not explained by pseudo-first order kinetic model. As shown in Table 3, a good agreement between calculated and experimental q_e_ values was observed and higher R^2^ values (0.9970–0.9994) were obtained. According to the previous studies, the adsorption kinetic data follows pseudo-second order when the boundary layer resistance is not the rate limiting step [44]. The phenomenon suggest that the rate controlling step was chemical adsorption which is driven by the electrostatic and π-π interactions between adsorbates molecules and the magnetic cellulose-chitosan adsorbent. These results are consistent with other literature reports [1,45,46].

To investigate the effect of magnetic cellulose-chitosan nanocomposite pore size on the adsorption process, the intraparticle diffusion model was applied to the kinetic data. The diffusion constants of the model can be calculated using Equation (3). Table 4 displays the intraparticle diffusion (K_di1_, K_di2_ and K_di3_) constants and the coefficient of determination (R^2^). The order of sorption for ATN, PRP, CBZ in the first step (K_di1_) was higher than in the second phase (K_di2_) and the third phase K_di3_, thus, the fitting of intra-particle diffusion model was separated into three phases for the adsorption of ATN, PRP, CBZ. The presence of two or more mass transfer mechanisms that control the adsorption process is a result of multilinearity [47]. The diffusion of adsorbate to the external surface of the adsorbent is the first stage resulting in the initial sharp increase in q_t_ [43,47]. The second and third phase is the intraparticle or pore diffusion stage which is rate limiting before finishing at a slower pace due to the approach to equilibrium [48]. This suggest that the last two stages may be attributed to a strong electrostatic attraction between the ATN, PRP, CBZ and the surface of the adsorbent [43].

### 2.6. Thermodynamics Studies

The thermodynamic parameters, that is, Gibbs free energy (ΔG°, kJ mol^−1^) enthalpy (ΔH°, kJ mol^−1^), and entropy (ΔS°, J mol^−1^ K^−1^) for the adsorption ATN, PRP and CBZ onto the surface of magnetic cellulose-chitosan nanocomposite were calculated according to the equations presented in Table S5. The adsorption thermodynamic studies were conducted at 298, 303 and 313 K and the results of the parameters are presented in Table 5. The thermodynamic results revealed that the values of ΔG° were negative suggesting that the adsorption process was spontaneous in nature. The values of ΔH° and ΔS° were positive indicating that the adsorption of ATN, PRP and CBZ was endothermic in nature with increased disorder at the interface. From Table 5, it can be noted that the values of ΔH° were greater than 20.9 kJ mol^−1^, which indicate that the adsorptions of the analytes was accredited to the chemical adsorption [49].

### 2.7. Application to Real Samples

#### 2.7.1. Occurrence of Atenolol, Propranolol, and Carbamazepine in Water Samples

Table 6 present the average concentration of ATN, PRP and CBZ detected in tested influent, effluent and river water. In influent wastewater, the average concentrations of ATN, PRP and CBZ were 1455 ng L^−1^, 201 ng L^−1^ and 894 ng L^−1^, respectively. As seen, the lowest concentration of PRP (0–83.4 ng L^−1^) was detected in river water samples while ATN and CBZ were almost in the same magnitude. The average concentrations of ATN, PRP and CBZ in effluent wastewater samples ranged from 457–1033 ng L^−1^, 116–176 ng L^−1^ and 406–537 ng L^−1^. These revealed that the investigated analytes were completely removed by the treatment processes as the water is discharged into the nearby river. The results obtained in this study were in line with those reported by [50].

#### 2.7.2. Removal Efficiency of Selected Pharmaceuticals

**Figure 10:**
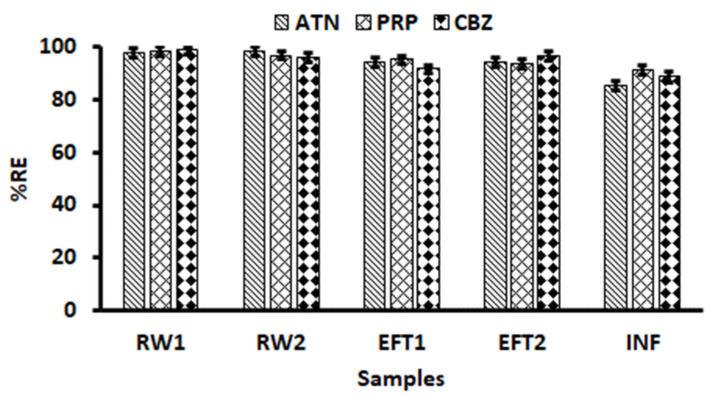
Adsorptive removal of ATN, PRP and CBZ from spiked river water (RW) and wastewater effluent (EFT) and influent (INF). Experimental conditions: mass of adsorbent = 54 mg, contact time = 34 min, sample volume = 5 mL, sample pH = 7.

The ability of the magnetic cellulose-chitosan nanocomposite to remove ATN, PRP and CBZ from real water samples (that is, river water upstream -RW 1, river water downstream- RW2, effluent (outflow)- EFT 1, effluent before chlorination and UV treatment -EFT 2 and influent) was examined. Since the concentrations present in the samples was low they were spiked with 2.0 mg L^−1^ of each analyte and the optimized method was applied. As seen in Figure 10, the percentage removal of ATN, PRP and CBZ ranged between 85–98%. These findings proved that the prepared nanocomposite retained high ATN, PRP and CBZ adsorption capacities for real water samples. Moreover, these results supported the evidence that the performance of the proposed magnetic cellulose-chitosan nanocomposite for the removal of ATN, PRP and CBZ can be anticipated as outstanding.

### 2.8. Regeneration Studies

**Figure 11:**
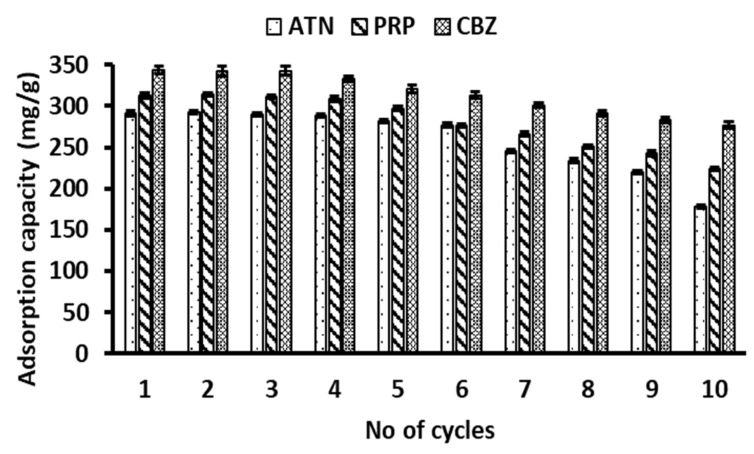
Regeneration studies for evaluation of the reusability of the magnetic cellulose-chitosan hydrogel.

To evaluate the reusability of the hydrogels, after sorption, the magnetic cellulose-chitosan nanocomposite was further regenerated with 100% of methanol and used for simultaneous sorption of (ATN, PRP and CBZ) solution of same concentration (200 mg/L) using the preconcentrating method. Repeated sorption-desorption experiments were carried out for ten consecutive cycles. The results in Figure 11 show that the adsorption capacity declined slightly as the number of adsorption-desorption cycles increased. Studies have reported that this phenomenon can be attributed to the chemisorption of pollutants as well as unavoidable weight loss of the hydrogel that occurs during the consecutive adsorption–desorption cycles [51,52]. More remarkably, even though the adsorbent lost 19–38% (retaining 61–81% adsorption capabilities) of its adsorption capacity after the tenth cycle, the removal efficiency was found to be greater than 80%, confirming that the prepared hydrogel material was recyclable sorbent for the target analytes. Even though the regeneration process used in this study seem to be effective, the major challenge is that the organic solvent used to recover the analytes from the adsorbent has a potential of caused secondary pollution. To overcome this drawback, advanced oxidation processes could be used for the regeneration of the adsorbent materials.

### 2.9. Comparison with Previous Studies

The adsorption capacities for the analytes (ATN, PRP, CBZ) were compared with other adsorbents based on previous findings from other authors, summarized in Table 7. Polymers showed effective adsorption capacity for ATN, PRP and CBZ compared to traditional adsorbents such as granular activated carbon, hematite nanoparticles and activated carbon fiber. Polymers have excellent adsorption properties due to their high surface area and favorable structural characteristics. Arya and Philip [53] tested the adsorptive removal of atenolol using synthesized magnetic polymer clay and the maximum adsorption capacity for atenolol was calculated to be 15.6 mg/g. The electrostatic activity was found to be the key driving force of adsorption, and the presence of humic acid affected the adsorbent performance at lower and higher pH levels. Similarly, Mhammed et al. [54] also studied the adsorptive removal of atenolol using a GO/PVP/AAc composite hydrogel, and it was found that the composite was effective in removing 90.2% in 90 min. In comparison, it was clearly shown that polymers have a good adsorption potential to remove ATN, PRP and CBZ from aqueous solution, this data is consistent with the results found in our study where the adsorption capacity for ATN, PRP and CBZ is 341 mg g^−1^, 313 mg g^−1,^ and 291 mg g^−1^, respectively.

## 3. Experimental

### 3.1. Material and Reagents

Analytical grade chemical reagents were use unless otherwise stated and double distilled water (Direct-Q^®^ 3UV-R purifier system Millipore Merck, Darmstadt, Germany) was used. Oxalic acid, microcrystalline cellulose (MCC), ammonium solution (NH_3_·H_2_O), chitosan (%), ferric chloride hexahydrate (FeCl_3_·6H_2_O), methanol (HPLC grade), acetonitrile (HPLC grade), ferrous chloride tetrahydrate (FeCl_2_·4H_2_O), propranolol hydrochloride (PRP, 99%), atenolol (ATN, 98%), carbamazepine (CBZ, 100%), glacial acetic acid, ethanol (EtOH) and sodium hydroxide (NaOH) were obtained from Sigma-Aldrich (St. Louis, MO, USA). Stock solutions of 1000 mg L^−1^ of each analyte, that is ATN, CBZ and PRP, were separately prepared by dissolving appropriate mass of target analytes in methanol. Synthetic sample mixtures composed of ATN, CBZ and PRP were freshly prepared by diluting appropriate volumes of stock solutions with ultra-pure water to a final volume of 100 mL to provide a concentration of 100 mg L^−1^. The stock solutions were stored in a refrigerator at 4–8 °C for further use.

### 3.2. Instrumentation

The morphology of the nano-adsorbent was analyzed by a 120 kV accelerating voltage transmission electron microscope (TEM, JEM-2100, JEOL, Tokyo, Japan) was used for sample analysis. Fourier transform infrared (FTIR) spectroscopy was used to analyze the sample spectra on a Spectrum 100 instrument (Perkin Elmer, Waltham, MA, USA). X-ray diffraction (XRD) patterns were obtained using PANAlytical XRD (PANalytical’s X’pert PRO, Almelo, The Netherlands). The adsorbent surface area and pore size distribution were recorded using an ASAP2020 porosity and surface area analyzer (Micrometrics Instruments, Norcross, GA, USA). An OHAUS starter 2100 pH meter (Pine Brook, NJ, USA) was used to record the pH of the solutions. A 5.7-L (internal dimensions: 300 × 153 × 150 mm) Scientech ultrasonic cleaner (Labotec, Midrand, South Africa) was used to promote adsorption. Vibrating sample magnetometry (VSM) (Cryogenic Ltd, London, UK) with temperature range of 1.8–320 K and magnetic field ±14 Tesla was used to measure the magnetic properties of the material.

### 3.3. Preparation of the Nanocomposite

#### 3.3.1. Synthesis of the Magnetic Cellulose

The method for synthesis of magnetic microcrystalline cellulose (MCC) is a modified version of the one reported by Xiong et al. [59]. Briefly 150 mg MCC, 0.5 g FeCl_3_·6H_2_O and 0.184 g FeCl_2_·4H_2_O were dissolved in 30 mL of ultrapure water and heated at 80 °C. Thereafter, 7.5 mL of ammonium hydroxide was added to the mixture with vigorous stirring black magnetic cellulose. The solution was vigorously stirred for further 30 min on a magnetic stirrer resulting in a magnetic composite of cellulose and iron oxide. The nanocomposite was collected using an external magnet and washed several times with deionized water. The resulting cellulose iron oxide was then oven dried at 50 °C overnight.

#### 3.3.2. Synthesis of the Magnetic Cellulose-Chitosan Hydrogel Nanocomposite

The magnetic cellulose-chitosan hydrogel nanocomposite was synthesized and modified using the method described in the literature by Mashile et al. [60] and Sharififard et al. [61]. Briefly, 5 g of chitosan was added to 500 mL solution of 0.2 mol L^−1^ oxalic acid under continuous stirring at 45–50 °C to form a viscous gel. The previously synthesized magnetic cellulose (5 g) was slowly applied to the chitosan gel and stirred at 45–50 °C for 2 h. A mixture of magnetic cellulose-chitosan gel was added dropwise to the 0.7 M NaOH precipitation bath to form beads. The formed beads were filtered from the NaOH bath and washed with deionized water several times until a neutral pH was achieved. Magnetic cellulose-chitosan formed beads were dried in the oven at 50 °C overnight and ground into fine powder with a mortar and pestle.

### 3.4. Ultrasound Assisted Batch Adsorption Studies

The batch adsorption experiments of ATN, CBZ and PRP from aqueous samples onto the magnetic cellulose-chitosan hydrogel nanocomposite were conducted using an ultrasonic bath. The ultrasonic power, frequency and heating system were set at 25 (±2) °C, 150 W and 50 kHz, respectively. The effect of three important independent parameters such as pH, contact time and mass of adsorbent were optimized using the central composite design (CCD). The independent variables were investigated at five levels and their actual values are presented in Table S2. Based on the design of experiments using CCD, the ultrasound-assisted batch adsorption was performed as follows: aliquots of 50 mL solutions (pH 2.4–7.6) containing a mixture of ATN, CBZ and PRP concentration level of 1.0 mg L^−1^ were placed in 100 mL sample bottles containing masses of adsorbent ranging from 15.7–54.3 mg. The samples were sonicated for 1.4–33.6 min at 25 ± 2 °C (ambient temperature). The adsorbent was separated from the sample solution via an external magnet and filtered through a 0.22 μm PVDF membrane syringe filter. Propanol, atenolol, and carbamazepine were measured using HPLC-DAD for the initial concentration and equilibrium. All the adsorption experiments were carried out in triplicates. Percentage removal efficiency (%RE) was used as the analytical response and it was calculated using Equation (1):

(1) $$\%\mathrm{RE}=\frac{C_{0}-C_{e}}{C_{0}}\times 100$$

where C_0_ and C_e_ are the initial and equilibrium concentrations (mg L^−1^) and the C_e_ concentration of the target analytes, respectively.

### 3.5. Adsorption Isotherms, Kinetics and Thermodynamic Experiments

To evaluate isotherms, kinetic models and thermodynamics, adsorption studies were performed. Synthetic solutions containing a mixture of atenolol, carbamazepine, and propranolol hydrochloride at different concentration were prepared ranging from 5 to 100 mg L^−1^, while other variables such as contact time and mass of adsorbent were fixed at optimal conditions. Kinetics adsorption studies were performed by the introduction of 50 mL of 100 mg L^−1^ atenolol, carbamazepine and propranolol hydrochloride solutions (pH 7.0) in 100 mL glass bottles containing 54 mg of magnetic cellulose-chitosan hydrogel nanocomposite. The sample solutions were agitated for 34 min by ultrasonication [62]. The effect of temperature on adsorption was studied by using various temperature that is, 20, 30, 40, and 50 °C with pH 7 and variable initial concentration (100 mg L^−1^).

### 3.6. Adsorption Data Analysis

#### 3.6.1. Adsorption Isotherms

Adsorption isotherm models play an important role in describing the types of adsorbent-adsorbent interactions that take place during the adsorptive removal process [61,63]. Adsorption isotherms provide assumptions with respect to the heterogeneity/homogeneity and interaction of adsorbent and adsorbate [63]. Equilibrium data was used to evaluate calculate the adsorption capacity of the adsorbent after the adsorption of propranolol hydrochloride, atenolol, and carbamazepine (Equation (2)):

(2) $$q_{e}=\frac{(C_{0}-C_{e})\;V}{M}$$

where q_e_ (mg g^−1^) is the quantity of propanol, atenolol, and carbamazepine taken up by magnetic cellulose-chitosan sorbent per gram, C_0_ and C_e_ (µg L^−1^) are the initial and equilibrium propranolol hydrochloride, atenolol, and carbamazepine concentrations, V (L) is the volume of the aqueous solution, M (g) is the mass of magnetic cellulose-chitosan. Adsorption isotherms such as Temkin, Redlich-Peterson, Langmuir and Freundlich models were used to explain the equilibrium data and their linearized equations are illustrated in Table S3.

#### 3.6.2. Adsorption Kinetic Models

Adsorption kinetics is one of the key factors that is used to investigate the efficiency adsorption process [41]. Therefore, linear equations of kinetics models such as Elovich, intraparticle diffusion, pseudo-first order and pseudo-second order, were used to interpret the adsorption data. These models were used to investigate the adsorption mechanism. The linearized equation for each model is illustrated in Table S4.

#### 3.6.3. Thermodynamics Studies

In order to determine the essence of the adsorption, the effect of temperature on the adsorption mechanism is studied by the measurement of thermodynamic properties. The design and viability of the magnetic cellulose nanocomposite adsorption cycle of hydrochloride propranolol, atenolol and carbamazepine using three main thermodynamic parameters including standard enthalpy (ΔH°), Gibbs free energy (ΔG°) and standard entropy (ΔS°) must be determined using the following equations displayed in Table S4. Thermosetting experiments were conducted at different temperatures, including 298, 308 and 318 K, by observing the adsorption mechanism. The equations in Table S5 were used to calculate the parameters [64,65]. 

### 3.7. Swelling Test

The magnetic cellulose-chitosan nanocomposite hydrogel percentage swelling ratio (%SR) was determined according to the method reported in the literature [66,67,68]. The experiments were carried out as follows: 54 mg of hydrogel nanocomposite was immersed in aqueous solutions at pH values ranging from 1–13 and it was incubated for 48 h [66]. The pH of the solutions was adjusted by the addition of 0.1 mol L^−1^ HCl and NaOH. Throughout the swelling process, the solution was changed periodically to ensure maximum equilibrium at the correct pH. The hydrogel percentage swelling ratio (SR%) was defined as follows:

(3) $$\mathrm{SR}\;\left[\%\right]=\frac{W_{s}-W_{d}}{W_{d}}\times 100$$

where W_s_ is the weight of the swollen hydrogels after the surface water has been absorbed with a wet filter paper, and W_d_ is the weight of dry hydrogel under ambient conditions [67,69,70].

### 3.8. Real Water Samples

Wastewater and river water samples were collected from a wastewater treatment plant in Daspoort (WWTP, Pretoria, South Africa) and the Apies River. Samples were collected in glass bottles and stored in the refrigerator (at 4 °C) until they were used. Before the removal process, the samples were filtered to remove particulates. The optimized procedure was used to remove ATN, PRP and CBZ from real wastewater samples.

### 3.9. Reusability Studies

The desorption of the pollutants adsorbed on the magnetic cellulose-chitosan hydrogel nanocomposite adsorbent was evaluated using methanol as the regenerator. To describe the method briefly, the hydrogel adsorbent (54 mg) was packed in 3 mL SPE empty columns and 100 mL sample solution containing ATN, CBZ and PRP each at 200 mg/L (25 °C, pH 7 was passed though the column. After reaching adsorption equilibrium, the analytes were eluted with 99.99% methanol and then the column washed with deionized water. The hydrogel adsorbent was reused in another the adsorption-desorption experiment. The sorption-desorption tests were performed were repeated ten times.

## 4. Conclusions

Our findings show that the prepared bio-adsorbent magnetic hydrogel nanocomposite possesses strong adsorption dynamics for propranolol hydrochloride, atenolol, and carbamazepine. pH 7.0 was defined as the optimal pH for the full adsorption and the RE is 98%. The hydrogel can be a suitable adsorbent for drug removal from wastewater. The experimental findings were modelled with isotherms of Langmuir and Freundlich. The Langmuir isotherm has been found to fit the data well. The hydrogel demonstrated that it could be used up to ten times while maintaining a removal efficiency of more than 80%. The specific characteristics and the availability of hydrogel make it an interesting, promising and environmentally friendly source for β-blockers and anticonvulsant adsorption in wastewater.

## Figures and Tables

**Table 1:** S_BET_, average pore size and total pore volume analysis data.

Surface Properties	Cellulose	Chitosan	Magnetic Cellulose	Nanocomposite
S_BET_ (m^2^ g^−1^)	1.53	1.58	75.3	112
Total pore volume (cm^3^ g^−1^)	0.46	0.36	0.39	0.59
Average pore size (nm)	21.2	12.8	20.7	24.1

**Table 2:** Adsorption isotherms of ATN, PRP and CBZ onto the magnetic cellulose-chitosan nanocomposite.

Isotherms	Parameters	Atenolol	Propranolol Hydrochloride	Carbamazepine
Langmuir	q_max_ (mg g^−1^)	341	313	291
	k_L_ (min ^−1^)	0.08	0.30	0.13
	R^2^	0.9939	0.9951	0.9942
Freundlich	K_F_	42.2	114	73.0
	N	1.7	3.8	2.9
	R^2^	0.9208	0.9836	0.9877
Sips	K_S_ (L mg^−1^)	0.07	0.34	0.27
	q_mS_ (mg g^−1^)	345	310	294
	n_S_	1.0	0.95	1.3
	R^2^	0.9956	0.9928	0.9907
Redlich–Peterson	β	1.2	0.95	0.98
	K_R-P_ (L g^−1^)	23.0	40.5	10.3
	α_R-P_	1.25	1.97	1.56
	R^2^	0.9925	0.9936	0.9917

**Table 3:** Adsorption kinetics of ATN, PRP and CBZ onto the magnetic cellulose-chitosan nanocomposite.

Kinetics	Parameters	Atenolol	Propranolol Hydrochloride	Carbamazepine
Experimental	q_e_ (mg g^−1^)	340	308	291
Pseudo-first order	q_e_ (mg g^−1^)	172	183	119
	k_1_	0.0899	0.0854	0.0929
	R^2^	0.8812	0.8720	0.8733
Pseudo-second order	q_e_ (mg g^−1^)	341	309	286
	k_2_	0.00078	0.0032	0.0013
	R^2^	0.9984	0.9970	0.9994

**Table 4:** Intraparticle diffusion model of ATN, PRP and CBZ onto the magnetic cellulose-chitosan nanocomposite.

Parameters	Atenolol	Propranolol Hydrochloride	Carbamazepine
K_id1_ (g mg^−1^ min^−0.5^)	41.6	33.4	31.7
C_1_	114	108	128
R^2^	0.9917	0.9983	0.9988
K_id2_ (g mg^−1^ min^−0.5^)	8.80	20.2	11.3
C_2_	284	184	220
R^2^	0.7669	0.8286	0.9077
K_id3_ (g mg^−1^ min^−0.5^)	0.295	0.301	0.056
C_3_	336	306	289
R^2^	0.9875	0.9877	0.6812

**Table 5:** Thermodynamics parameters for the adsorption of ATN, PRP and CBZ.

Analytes	Temp (K)	ΔG° (kJ mol^−1^)	ΔH° (kJ mol^−1^)	ΔS° (J mol^−1^ K^−1^)
Atenolol	298	−4.28	61.9	21.3
	303	−4.40		
	308	−4.48		
	313	−4.61		
Propranolol	298	−4.08	77.3	22.3
	303	−4.20		
	308	−4.31		
	313	−4.41		
Carbamazepine	298	−3.82	84.7	31.4
	303	−3.96		
	308	−4.11		
	313	−4.30		

**Table 6:** Concentrations (ng L^−1^) of ATN, PRP and CBZ detected in real water samples.

Samples	Atenolol	Propranolol	Carbamazepine
River water upstream	105 ± 2	ND	103 ± 4
River downstream	116 ± 3	83.4 ± 2	167 ± 3
Effluent (outflow)	457 ± 4	116 ± 3	406 ± 6
Effluent before chlorination and UV treatment	1033 ± 11	176 ± 2	537 ± 7
Influent	1455 ± 15	201 ± 3	894 ± 10

**Table 7:** Comparison of adsorption capacity of the present pharmaceuticals with other reported pharmaceuticals.

Analytes	Adsorbent	Adsorption Capacity (mg/g)	References
Propranolol	Smectite clay mineral montmorillonite	161	[55]
Carbamazepine and Propranolol	Activated carbon fiber	(0.300 ± 0.014, 0.277 ± 0.021	[56]
Atenolol	Granular activated carbon	1.2	[57]
Atenolol and Carbamazepine	Activated palm kernel shell	0.184 and 0.170	[43]
Carbamazepine	Hematite nanoparticles	2.89	[4]
Carbamazepine	β-cyclodextrin polymer	136.4	[58]
Atenolol	Magnetic polymer clay composite	15.6	[53]
Atenolol	GO/PVP/AAc composite hydrogel	0.0107	[54]
Carbamazepine, Propranolol and Atenolol	Magnetic cellulose-chitosan nanocomposite	291, 313 and 341	This study

## Supplementary Materials

The following are available online at https://www.mdpi.com/article/10.3390/gels7040190/s1, Table S1: Central composite design matrix and respective analytical response; Table S2: Central composite design variables and their levels; Table S3: Propranolol hydrochloride, atenolol, and carbamazepine adsorption isothermal parameters; Table S4: Kinetics models ad linearized equations; Table S5: Thermodynamics equations and expressions.
