# Supplementary material for: Magnetic Cellulose-Chitosan Nanocomposite for Simultaneous Removal of Emerging Contaminants: Adsorption Kinetics and Equilibrium Studies
Source: Gels. 2021 Oct 30;7(4):190. doi: 10.3390/gels7040190 (PMC8628732; doi:10.3390/gels7040190)
Supplement: Supplementary file 1 [file gels-07-00190-s001.zip › gels-1416796-supplementary.pdf]

Magnetic Cellulose-Chitosan Nanocomposite for Simultaneous Removal of Emerging Contaminants: Adsorption Kinetics and Equilibrium Studies

Phodiso P. Mashile [1,2], Philiswa N. Nomngongo [1,2*]

[1] Department of Chemical Sciences, University of Johannesburg, Doornfontein Campus, P.O. Box 17011, Doornfontein 2028, South Africa; prudencem.mashile@gmail.com

[2] Department of Science and Innovation-National Research Foundation South African Research Chair
Initiative (DSI-NRF SARChI): Nanotechnology for Water, University of Johannesburg, Doornfontein 2028, South Africa

* Correspondence: pnnomngongo@uj.ac.za

Table S1 Central composite design matrix and respective analytical response

**Standard Run**	**Parameters**			**Atenolol**	**Propanol**	**Carbamazepine**
	ET	MA	pH		%RE	
**1**	5.0	20.0	3.0	29.8	30.5	34.4
**2**	5.0	20.0	7.0	74.0	63.3	65.3
**3**	5.0	50.0	3.0	52.7	62.0	53.5
**4**	5.0	50.0	7.0	81.6	78.0	69.8
**5**	30.0	20.0	3.0	69.1	67.5	71.0
**6**	30.0	20.0	7.0	99.5	97.1	98.1
**7**	30.0	50.0	3.0	81.7	70.8	80.1
**8**	30.0	50.0	7.0	97.7	98.4	99.1
**9**	1.4	35.0	5.0	10.2	15.2	16.2
**10**	33.6	35.0	5.0	80.6	70.6	89.0
**11**	17.5	15.7	5.0	73.9	53.6	77.9
**12**	17.5	54.3	5.0	80.7	73.2	93.5
**13**	17.5	35.0	2.4	22.3	22.7	25.4
**14**	17.5	35.0	7.6	94.8	94.9	93.8
**15 (C)**	17.5	35.0	5.0	77.5	76.3	80.0
**16 (C)**	17.5	35.0	5.0	79.9	74.0	82.4

Table S2 Central composite design variables and their levels

**Variables**	**-α (-1.28)**	**Low level (-1)**	**Central point (0)**	**High level (1)**	**α (1.28)**
Sample pH	2.43	3	5	7	7.57
Mass of adsorbent (MA), mg	15.7	20	35	50	54.3
Contact time (CT) Min	1.41	5	17.5	30	33.6

Table S3 Propranolol hydrochloride, atenolol, and carbamazepine adsorption isothermal parameters

Isotherm models	Isotherm expression	Definition of terms
Langmuir	$\frac{C_e}{q_e} = \frac{1}{q_{\max} K_L} + \frac{C_e}{q_{\max}}$	q_{max}: theoretical monolayer adsorption capacity (mg g^{-1})
	$R_L = \frac{1}{1 + K_L C_0}$	C_e: equilibrium concentration (mg L^{-1}), q_e: the amount of adsorbate adsorbed per unit weight of adsorbent (mg g^{-1})
		C_0: initial concentration (mg g^{-1})
		K_L: Langmuir equilibrium constant (L mg^{-1})
		R_L: separation factor
Freundlich	$\ln q_e = \ln K_F + \ln C_e$	K_F: Freundlich constant (L g^{-1})
		n: is the Freundlich exponent (g L^{-1})
Sips	$q_e = \frac{q_{max} K_{eq} C_e^n}{1 + K_{eq} C_e^n}$	K_{eq}: equilibrium constant (L/mg)
		q_{max}: maximum adsorption capacity (mg/g)
		n: heterogeneity factor
Redlich-Peterson	$1n\left(A\frac{C_e}{q_e} - 1\right) = g\ 1nC_e + 1nB$	A,g, and B : Redlich- Peterson constants
		C_e: adsorbate concentration (mg/L) remaining in the solution
		q_e: adsorption capacity (mg/g) of hydrogels at equilibrium

Table S4 Kinetics models ad linearized equations

Kinetic models	Kinetic Expression	Definitions of terms
Pseudo-first order	$In(q_e - q_t) = \ln q_e - k_i t$	K_1: rate constant(min^{-1})<br>q-q_e: amount of absorbate at equilibrium (mg g^{-1})
Pseudo-second order	$\frac{1}{q_t} = \frac{1}{k_2 q_e{}^2} + \frac{1}{q_e} t$	K_2: Equilibrium rate constant (g.mg^{-1} min^{-1})<br>q_t and q_e: amount of adsorbent at equilibrium (mg g^{-1})
Intraparticle	$Q_t = k_i t^{\frac{1}{2}} + C$	Q_t: amount of solute on surface of sorbent at time t (mg g^{-1})<br>K_i: intraparticle diffusion constant (mg g^{-1} min$^{1/2}$)

Table S5 Thermodynamics equations and expressions

Equations	Equation expression	Definitions of terms
**1**	$\Delta G = -RT\ln K_L$	R: is the universal gas constant (8.314 J mol^{-1} K^{-1})
		T: is the temperature
		K_L: is the Langmuir constant (L mol^{-1})
**2**	$\Delta G = \Delta H - T\Delta S$	T: is the temperature
		ΔH and ΔS: values were estimated from the intercept and slope of a plot of ΔG versus T
**3.**	lnK = $lnK = -\frac{\Delta H°}{RT} + \frac{\Delta S°}{R}$	T: is the absolute temperature (K),
		R: is the universal gas constant (8.314 J/mol-K)
		K: separation factor, $K = \frac{q_e}{C_e}$

The Gibbs free energy represents the spontaneous (ΔG° > 0) or non-spontaneous (DG °< 0) adsorption while the standard enthalpy reveals the endothermic (ΔH° > 0) or exothermic (DH° < 0) nature of adsorption process and standard entropy which corresponds to increased (ΔS° > 0) or decreased (ΔS° < 0) randomness at the solid-liquid interface upon adsorption (Mousavi et al., 2018).
